# Application of intravoxel incoherent motion imaging in the diagnosis of polycystic ovary syndrome

**DOI:** 10.3389/fmed.2025.1513710

**Published:** 2025-06-05

**Authors:** Keying Wang, Qian Song, Anrong Zeng, Ying Li, Jinwei Qiang

**Affiliations:** Department of Radiology, Jinshan Hospital of Fudan University, Shanghai, China

**Keywords:** polycystic ovary syndrome, magnetic resonance imaging, intravoxel incoherent motion, diffusion weighted imaging, diagnosis

## Abstract

**Objective:**

To evaluate the diagnostic efficacy of intravoxel incoherent motion (IVIM) parameters in ovarian stroma changes in polycystic ovary syndrome (PCOS) patients.

**Methods:**

This prospective study included 58 PCOS patients and 60 healthy controls. Both groups underwent conventional magnetic resonance imaging (MRI) and IVIM scans. The diagnostic efficacy of IVIM parameters and conventional MRI was investigated and compared by using area under the curves (AUC). Binary logistic regression analysis, integrated discrimination improvement (IDI), and net reclassification improvement (NRI) were also used to assess the improvement in diagnosis of PCOS when combining IVIM parameters with conventional MRI features.

**Results:**

The apparent diffusion coefficient (ADC) and D values were significantly lower, while the D* and *f* values were significantly higher than the controls in the ovarian stroma in PCOS patients. The follicle number (FN) counted on the MRI showed an AUC of 0.90 (95%CI: 0.84–0.96) for differentiating PCOS from the controls, with a cutoff value of 23. The ADC value yielded the highest AUC of 0.83 (95%CI: 0.76–0.90) among the IVIM parameters, with a cutoff value of 1.04 × 10^−3^ mm^2^/s. The diagnostic performance was significantly by combining ADC and FN with an AUC of 0.94 (95%CI: 0.90–0.98). The IDI and NRI also showed improved diagnostic performance than FN alone.

**Conclusion:**

The D* and *f* values of the ovarian stroma in PCOS patients are significantly increased, while the ADC and D values are significantly reduced. The combination of ADC and FN can improve the diagnostic efficacy for PCOS.

## Introduction

Polycystic ovary syndrome (PCOS) is a common endocrine disorder characterized by ovarian dysfunction, obesity, infertility, and hyperandrogenism (1). PCOS is a significant cause of female infertility, with a prevalence rate ranging from 6 to 20% (2). Beyond reproductive issues, PCOS significantly increases the risk of developing type 2 diabetes, obesity, endometrial cancer, hypercholesterolemia, and cardiovascular diseases (3). Therefore, early and accurate diagnosis is essential for effective management and prevention of long-term complications. Current imaging techniques play a central role in diagnosing PCOS, typically evaluating features such as increased follicle number (FN) and ovarian volume (OV) using ultrasound (US) or magnetic resonance imaging (MRI) (4, 5).

Compared to US, MRI offers superior soft tissue contrast and multiplanar imaging capabilities, which provides a more comprehensive view of ovarian structure (6). Recent studies have also highlighted the relevance of ovarian stromal characteristics-such as stromal volume, blood flow, and echogenicity distribution-in PCOS pathophysiology (7). Ovarian stroma is a major structural component of the ovary and accounts for a significant proportion of its volume. However, these stromal features remain underexplored in routine diagnostic evaluations.

Diffusion-weighted imaging (DWI) is a functional MRI technique that quantifies water molecule diffusion through the apparent diffusion coefficient (ADC) values, which can reflect microstructural changes in ovarian stroma (8). Samanci et al. found significantly lower ADC values in PCOS patients compared to controls by using a mono-exponential model of DWI. A moderate sensitivity of 78% and specificity of 62.9% was shown for diagnosing PCOS (9). However, ADC measurements can be confounded by both tissue diffusion and microcirculation, which may limit the diagnostic accuracy. Intravoxel incoherent motion (IVIM) is a novel functional imaging technique based on bi-exponential model. IVIM separates pure molecular diffusion from microcirculatory perfusion effects, which offers more detailed insight into tissue physiology (10).

We hypothesized that IVIM-derived parameters can better characterize ovarian stromal alterations and microcirculatory changes in PCOS. Therefore, this study aims to evaluate the diagnostic performance of IVIM in PCOS by comparing ovarian morphological features and IVIM parameters between PCOS patients and healthy controls. Our goal is to investigate the relationship between ovarian stromal changes and IVIM parameters in PCOS patients, and to evaluate IVIM’s potential as a non-invasive diagnostic tool for PCOS.

## Materials and methods

### Ethical statement

This prospective study was approved by the Review Board of Local Hospital (Approval No.: JIEC-2019-S31), and written informed consent was obtained from all participants.

### Study design and general information

The study included 58 consecutive PCOS patients from the Outpatient Clinic of Gynecology and Endocrinology and 60 healthy volunteers from the Health Management Center between December 2019 and December 2020. Clinical basic data, laboratory results, and imaging data were collected to compare ovarian MRI morphological features and IVIM parameters between the two groups. The correlation between ovarian morphological and stromal changes in PCOS patients was investigated, and the diagnostic efficacy of IVIM for PCOS was evaluated.

The diagnostic criteria for PCOS were as follows, with a diagnosis confirmed if at least two of the three conditions were met: (1) oligo- or anovulation (OA). (2) Clinical or biochemical hyperandrogenism (HA). (3) polycystic ovaries (PCO) on US.

The exclusion criteria were as follows: (1) Ovulation disorders related to other diseases, including thyroid dysfunction and hyperprolactinemia. (2) Hyperandrogenism caused by adrenal hyperplasia, severe insulin resistance syndrome, or androgen-secreting tumors. (3) Idiopathic hyperandrogenism or hirsutism. (4) Presence of corpus luteum, cysts, or dominant follicles.

### Clinical data

Clinical information for each subject, including age, menstrual status, physical examination results, and body mass index (BMI = weight/height^2^) were recorded. Approximately 10 mL of venous blood was drawn for clinical laboratory tests, including measurements of free testosterone (T), luteinizing hormone (LH), and follicle-stimulating hormone (FSH). LH/FSH was calculated. For PCOS patients with regular menstrual cycles and for control subjects, venous blood was collected on the 3rd to 5th day of the menstrual cycle. For PCOS patients with irregular menstrual cycles, blood was drawn at any time during the cycle. The control group underwent clinical laboratory and imaging examinations on the same day, while the interval between clinical laboratory tests and imaging examinations for PCOS patients was kept to less than 15 days.

### US examination

A 2D US (Philips HD11, 6.0 MHz, Netherlands) was used for patient examination. Transvaginal US was performed unless contraindicated, in which case abdominal US was used. PCO was diagnosed by transvaginal US if there were ≥12 follicles (2–9 mm in diameter) or if ovarian volume exceeded 10 cm^3^ in any ovary; abdominal US diagnosed PCO if any ovary had a volume greater than 10 cm^3^. Women with regular menstrual cycles were examined during the early follicular phase (days 3–5 of the menstrual cycle). If a dominant follicle (>10 mm) or corpus luteum was present, re-examination was performed in the next cycle. For those with irregular cycles or amenorrhea, scanning was conducted at any time during the cycle.

### MRI examination

MRI scans were performed using a 3.0 T MRI scanner (Magneton Verio, Siemens, Erlangen, Germany) with a pelvic phased-array coil. Scans for PCOS patients with regular menstrual cycles and for the control participants were conducted on days 3–5 of the menstrual cycle. For those with irregular cycles, scans were performed at any time during the cycle.

All subjects fasted for 4–6 h before the MRI scan, lay supine, and practiced calm thoracic breathing during the scan. The scan range extended from the anterior superior iliac spine to the upper femur. Routine MRI sequences were performed, including axial, coronal, and sagittal T2-weighted imaging (T2WI), followed by axial IVIM sequences. Single-shot spin-echo echo-planar imaging (EPI) sequences were used with 8 b-values (0, 50, 100, 150, 200, 400, 600, 1,000). The scanning sequences and parameters are listed in Supplementary Table 1.

### MRI image post-processing

A radiologist (Radiologist 1, with 7 years of experience in gynecological imaging) blinded to the clinical and laboratory results, observed and measured the MRI images. The OV was calculated using the T2WI sequence with the formula: OV = 0.523 × length × width × thickness. The FN was recorded as the average value of the left and right ovaries on T2WI sequence. To assess reproducibility, 30 images were randomly selected, and Radiologist 1 and Radiologist 2 (with 10 years of experience in gynecological imaging) remeasured follicle count and OV and redrew the region of interest (ROI) one month later. Interclass and intraclass correlation coefficients (ICC) were used to evaluate intra- and inter-observer reproducibility.

IVIM sequences were post-processed using Siemens software to automatically generate ADC maps. The original IVIM images were imported into the MATLAB software package (Version 7.9; Mathworks, Natick, Mass, USA) to obtain pseudo-color maps of the true diffusion coefficient (D), pseudo-diffusion coefficient (D*), and perfusion fraction (f). To reduce artifacts and calculation errors, an upper limit for D* was set at 170 s/mm^2^ based on the settings of Siemens IVIM post-processing software. The ROI for the stroma was drawn on the plane showing maximum ovarian stroma on the IVIM image, using the corresponding T2WI for reference. This process was repeated three times, and the average values for ADC, D, D*, and f were recorded.

### Diagnostic efficacy of MRI and IVIM for differentiating PCOS

Parameters with significant differences between the POCS and control groups were evaluated for diagnostic efficacy using receiver operating characteristic (ROC) curves. The DeLong test was used to compare the area under the curve (AUC). Net reclassification improvement (NRI) and integrated discrimination improvement (IDI) were calculated to determine whether the combination of IVIM functional parameters with morphological parameters significantly enhanced diagnostic efficacy.

### Data analysis

Statistical analysis of ovarian morphological parameters (OV, FN) and functional imaging parameters (ADC, D, D*, f) for the PCOS and control groups was performed using R software (version 4.4.0; http://www.R-project.org). Initially, normality tests were applied to the quantitative parameters. Independent sample t-tests were employed for data that followed a normal distribution, while the Mann–Whitney U test was used for data that did not meet this criterion. Chi-square tests were utilized for analyzing qualitative data. Pearson correlation coefficients were used calculated to examine the relationship between IVIM functional parameters and morphological parameters. The data were presented as mean ± standard deviation or N (%). A *p*-value of less than 0.05 was considered statistically significant.

## Results

### General clinical characteristics

**Figure 1:**
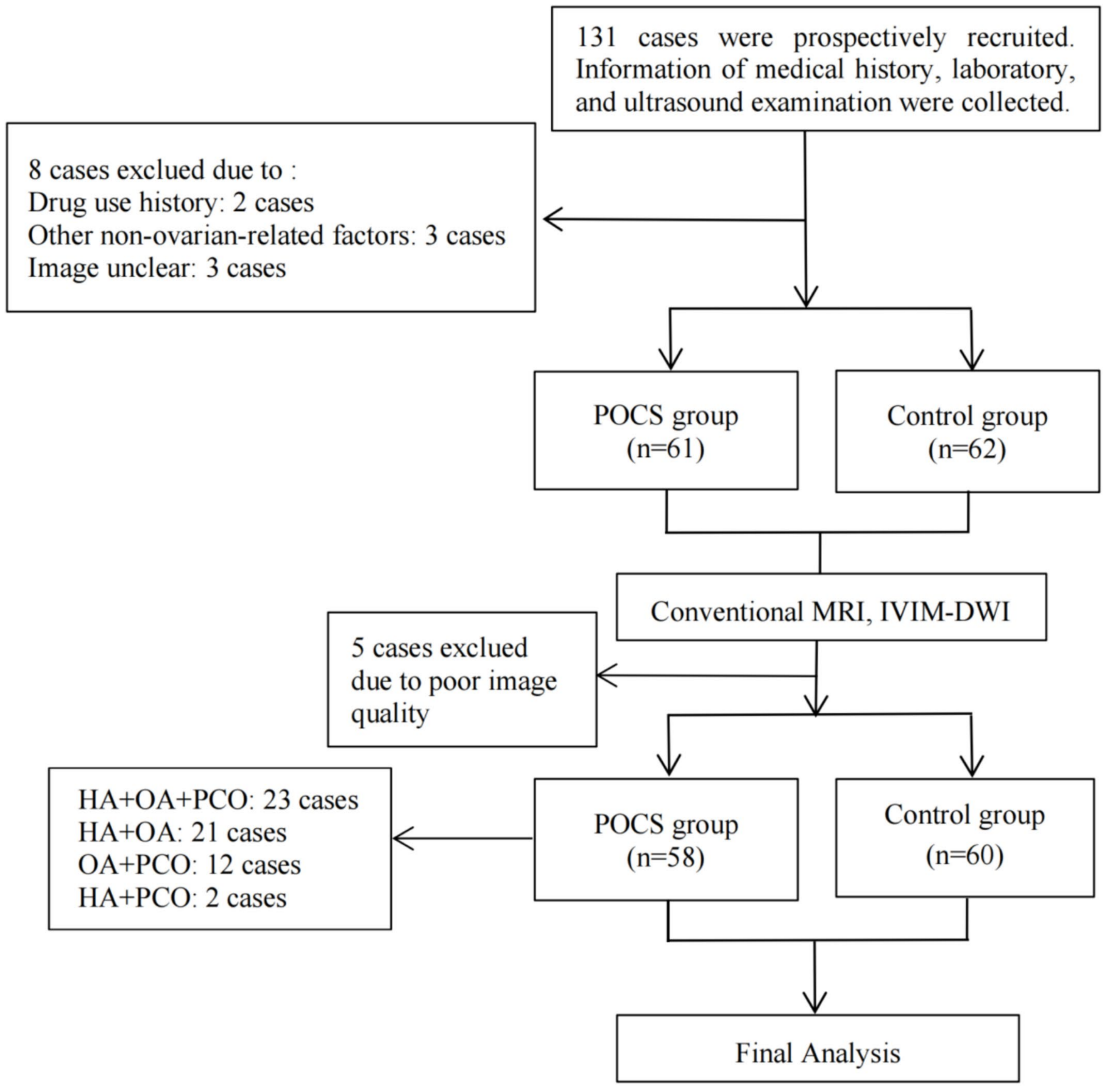
Study flowchart. The flowchart outlines the process of subject recruitment, data collection, and analysis for the study. POCS, polycystic ovary syndrome; MRI, magnetic resonance imaging; IVIM-DWI, intravoxel incoherent motion- diffusion weighted imaging; OA, oligo- or anovulation; HA, hyperandrogenism; PCO, polycystic ovaries.

The study flowchart is shown in Figure 1. Initially, 131 subjects were enrolled in the study; however, 13 were excluded. As a result, 118 subjects were analyzed in the final analysis. All subjects were aged 18 to 36 years (25 ± 3.5 years) and were between menarche and menopause. None of the subjects were pregnant or under any medication at the time of examination. 58 subjects were in the PCOS group and 60 were in the control group. The general clinical characteristics are presented in Table 1.

**Table 1:** Clinical characteristics of the PCOS group and control group.

Clinical and imaging feature	Control	PCOS	*P*-value
	(*N* = 60)	(*N* = 58)	
Age (y)	26 ± 2.5	25 ± 4.9	0.301
BMI (kg/m^2^)	20 ± 3.0	24 ± 4.5	< 0.001
OV (cm)	7.0 ± 2.5	12.2 ± 4.4	< 0.001
FN	14 ± 4.6	27 ± 9.2	< 0.001
ADC (×10^−3^ mm^2^/s)	1.26 ± 0.23	1.01 ± 0.16	< 0.001
D (×10^−3^ mm^2^/s)	1.36 ± 0.20	1.20 ± 0.15	< 0.001
D* (×10^−3^ mm^2^/s)	9.48 ± 1.72	10.50 ± 1.09	< 0.001
f	0.13 ± 0.06	0.15 ± 0.04	0.006
LH (IU/L)	5.5 ± 2.9	12.4 ± 7.9	< 0.001
FSH (IU/L)	8.0 ± 1.6	6.2 ± 1.9	< 0.001
T (ng/mL)	0.4 ± 0.1	0.8 ± 0.4	< 0.001
LH/FSH	0.7 ± 0.4	2.0 ± 1.1	< 0.001
PCO-US			< 0.001
Negative	50 (83.3%)	22 (37.9%)	
Positive	10 (16.7%)	36 (62.1%)	
Hyperandrogenism			< 0.001
Negative	51 (85.0%)	12 (20.7%)	
Positive	9 (15.0%)	46 (79.3%)	
Oligoanovulation			<0.001
Negative	51 (85.0%)	2 (3.4%)	
Positive	9 (15.0%)	56 (96.6%)	

BMI, body mass index; FN, follicle counted on MRI; OV, ovarian volume calculated on MRI; PCO-US, polycystic ovary observed by ultrasound; ADC, apparent diffusion coefficient; D, true diffusion coefficient; D*, pseudo-diffusion coefficient; f, perfusion fraction; LH/FSH, luteinizing hormone/follicle stimulating hormone; IVIM, intravoxel incoherent motion; PCOS, polycystic ovary syndrome; T, testosterone.

### Subgroup analysis

The specific subtypes in the PCOS group were as follows: 22 cases of HA + OA + PCO, 22 cases of HA + OA, 12 cases of OA + PCO, and 2 cases of HA + PCO (Supplementary Table 2). The age was similar across all groups with no statistically significant difference observed. However, BMI was significantly higher in HA + OA, HA + OA + PCO and OA + PCO groups compared to controls.

OV and FN were both significantly elevated in the HA + OA, HA + OA + PCO and OA + PCO groups compared to controls. LH levels were markedly elevated in the HA + OA and HA + OA + PCO group. FSH levels were lower in all these groups, notably in HA + OA + PCO, resulting in a significantly higher LH/FSH ratio across all the groups. Testosterone levels were significantly elevated in the HA + OA and HA + OA + PCO groups compared to controls.

IVIM showed significantly reduced ADC and D values in the HA + OA, HA + OA + PCO and OA + PCO groups. D* values were significantly reduced in HA + OA + PCO group but f did not show statistically significant differences in all groups.

### MRI ovarian morphological characteristics and diagnostic efficacy

**Figure 2:**
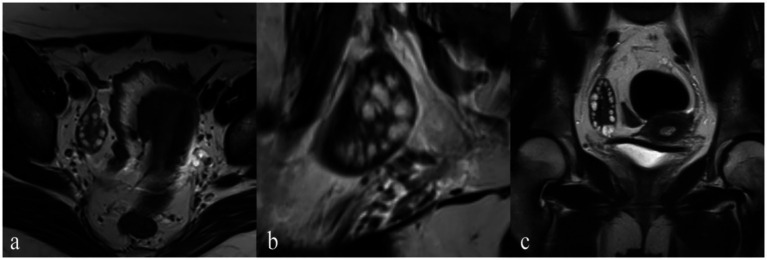
A PCOS patient with amenorrhea, acne, and hirsutism (Ferriman-Gallwey Score: 12). The patient has 36 follicles, and the ovarian volume is 22 cm^3^. **(a)** Axial T2-weighted imaging (T2WI) provides a cross-sectional view emphasizing the distribution of the follicles. **(b)** Sagittal T2WI displays the ovaries with multiple follicles. **(c)** Coronal T2WI displays the view highlighting the follicles’ arrangement and size.

Figure 2 illustrates the MRI scans of PCOS patients along with the volume calculation. The OV and FN in PCOS patients were significantly higher than those in the control group as observed on conventional MRI (both *p* < 0.001). The AUC for OV measured by MRI was 0.85 (95% CI: 0.78–0.93), with a sensitivity of 69% and a specificity of 93% (Table 2). The AUC for total FN was 0.90 (95% CI: 0.84–0.96), with a sensitivity of 72% and a specificity of 97% (Table 2).

**Table 2:** Diagnostic efficacy of conventional MRI and IVIM parameters.

Parameter	AUC (95% CI)	Cutoff value	Specificity	Sensitivity	NPV	PPV
OV (cm^3^)	0.85 (0.78–0.93)	10.7	0.93	0.69	0.76	0.91
FN	0.90 (0.84–0.96)	23	0.97	0.72	0.78	0.95
ADC (×10^−3^ mm^2^/s)	0.83 (0.76–0.90)	1.04	0.87	0.66	0.72	0.83
D (×10^−3^ mm^2^/s)	0.79 (0.70–0.87)	1.29	0.68	0.78	0.76	0.70
D* (×10^−3^ mm^2^/s)	0.74 (0.65–0.83)	9.21	0.57	0.88	0.83	0.66
f	0.71 (0.61–0.80)	0.12	0.52	0.81	0.74	0.62
Combined (ADC + FN)	0.94 (0.90–0.98)	0.49	0.95	0.86	0.88	0.94

NPV, negative predictive value; PPV, positive predictive value; AUC, area under the curve; MRI, magnetic resonance imaging.

### IVIM parameters and their diagnostic efficacy

**Figure 3:**
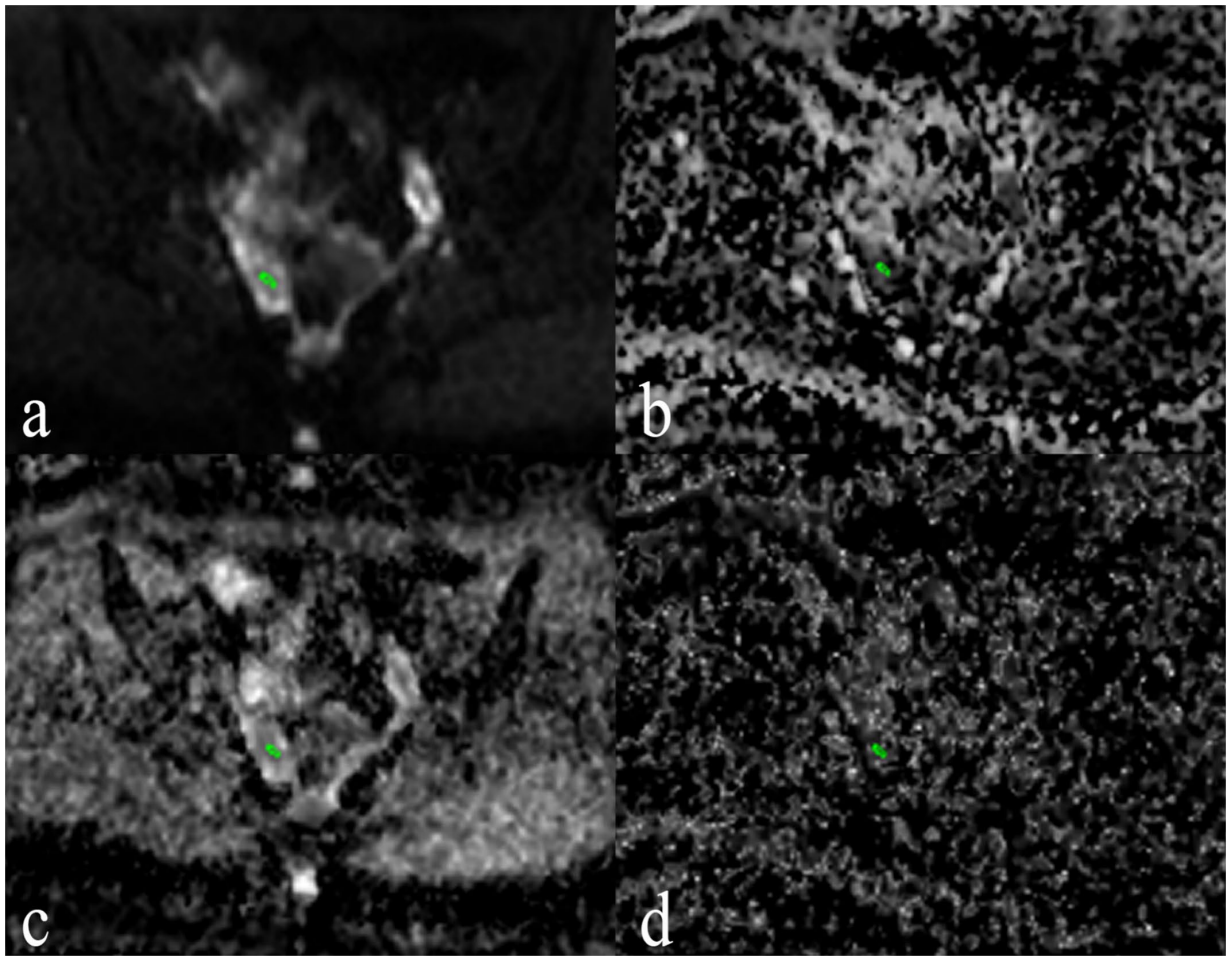
Post-processed IVIM sequence images of a PCOS Patient. **(a)** Apparent diffusion coefficient (ADC) Map provides the ADC values, reflecting overall water molecule mobility and tissue microstructure. **(b)** f Map provides the perfusion fraction values, reflecting the microvascular density within the ovarian stroma. **(c)** D Map provides the true diffusion coefficient values, representing the diffusion of water molecules within the tissue. **(d)** D* Map provides the pseudo -diffusion coefficient values, indicating perfusion-related incoherent motion within the tissue.

Figure 3 presents post-processed IVIM sequence images of PCOS patients. The ADC and D values of the ovarian stroma in PCOS patients were significantly lower than those in the control group (both *p* < 0.001) (Table 1). The cutoff value for ADC was 1.04 × 10^−3^ mm^2^/s, with a sensitivity, specificity, and AUC of 66, 87%, and 0.83 (95% CI: 0.76–0.90), respectively (Table 2). The cutoff value for D was 1.29 × 10^−3^ mm^2^/s, with a sensitivity, specificity, and AUC of 78, 68%, and 0.79 (95% CI: 0.70–0.87), respectively (Table 2). The D* and *f* values of the ovarian stroma in PCOS patients were significantly higher than those in the control group (*p* < 0.001 and *p* = 0.006) (Table 1). The cutoff value for D* was 9.21 × 10^−3^ mm^2^/s, with a sensitivity, specificity, and AUC of 88, 57%, and 0.74 (95% CI: 0.65–0.83), respectively (Table 2). The cutoff value for f was 0.12 × 10^−3^ mm^2^/s, with a sensitivity, specificity, and AUC of 81, 52%, and 0.71 (95% CI: 0.61–0.80), respectively (Table 2).

Reproducibility tests for ADC, D, D*, and f values demonstrated good intra- and inter-observer ICCs: for ADC [0.83 (95% CI: 0.76–0.90) and 0.79 (95% CI: 0.70–0.87)], for D [0.84 (95% CI: 0.67–0.92) and 0.83 (95% CI: 0.66–0.92)], for [D* 0.81 (95% CI: 0.60–0.91) and 0.74 (95% CI: 0.45–0.87)], and for f [0.85 (95% CI: 0.70–0.93) and 0.89 (95% CI: 0.77–0.95)].

The combination of ADC value and FN for distinguishing between PCOS patients and controls resulted in a combined diagnostic AUC of 0.94 (95% CI: 0.90–0.98), with an NRI of 0.19 (95% CI: 0.07–0.30; *p* = 0.001) and an IDI of 0.12 (95% CI: 0.07–0.17; *p* < 0.001) compared to FN along. However, the combined diagnostic AUC for D, D*, and f with FN did not show significant improvement compared to FN alone, with NRI and IDI of 0.10 (95% CI: 0.01–0.21) and 0.07 (95% CI: 0.02–0.12) for D, 0.03 (95% CI: 0.04–0.11) and 0.06 (95% CI: 0.02–0.11) for D*, and 0.05 (95% CI: 0.03–0.13) and 0.03 (95% CI: 0.01–0.06) for f.

### Correlation between IVIM and morphological parameters

**Figure 4:**
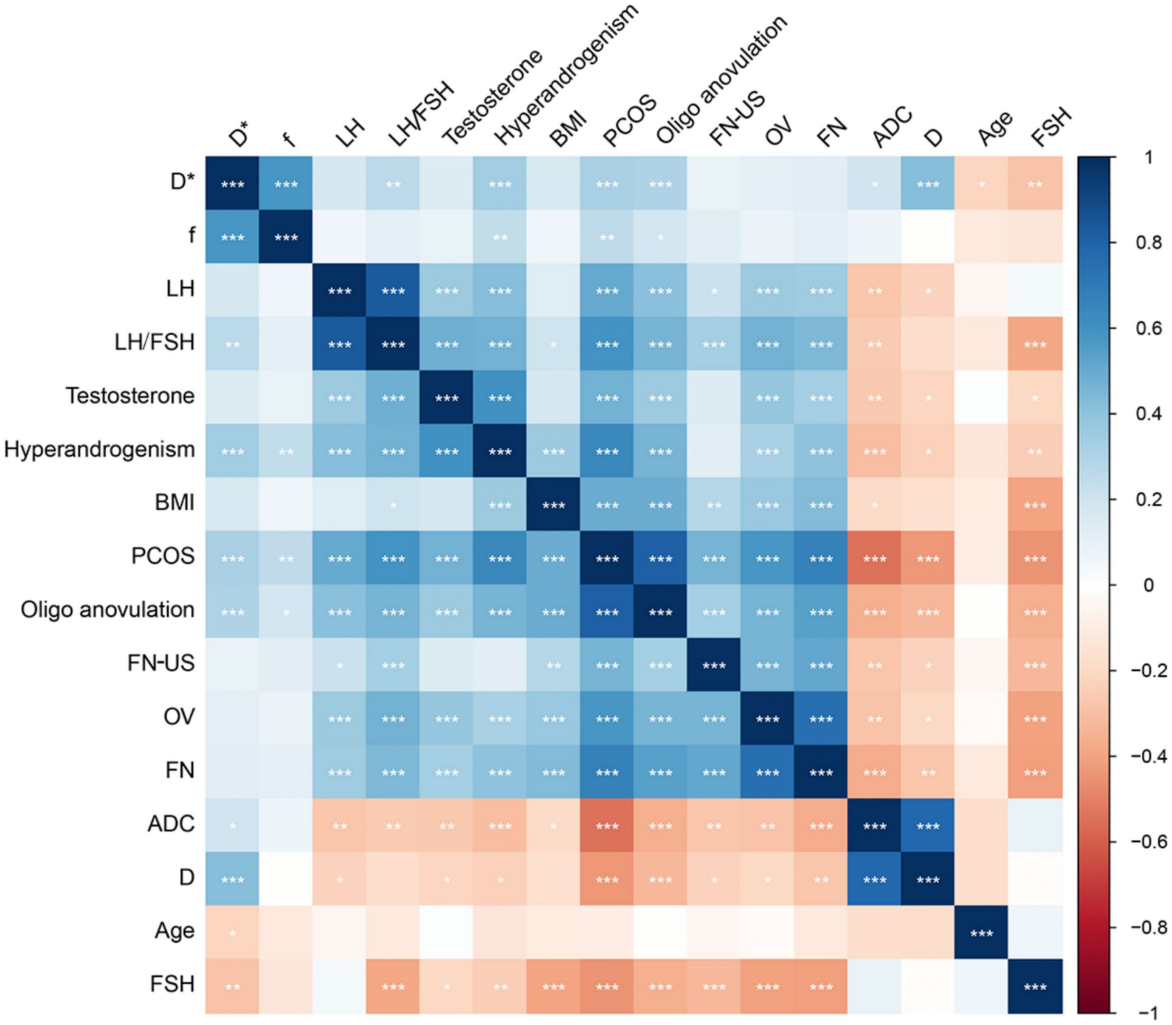
Correlation heatmap between morphological parameters and functional parameters. The color intensity in the heatmap indicates the strength and direction of the correlation between the parameters. Darker colors typically represent stronger correlations, whether positive (blue) or negative (red). Asterisks on the heatmap cells denote the significance of the correlation. ^*^p < 0.05; ^**^p < 0.01; ^***^p < 0.001. BMI, body mass index; FN, follicle counted on MRI; OV, ovarian volume calculated on MRI; FN-US: follicle count by ultrasound; ADC: apparent diffusion coefficient; D, true diffusion coefficient; D*, pseudo-diffusion coefficient; f: perfusion fraction; LH, luteinizing hormone; FSH, follicle stimulating hormone; IVIM, intravoxel incoherent motion; PCOS, polycystic ovary syndrome.

The heatmap of the correlation between morphological parameters and IVIM functional parameters revealed that ADC and D values were negatively correlated with morphological parameters, whereas D* and *f* values showed no significant correlation with morphological parameters (Figure 4). In both the PCOS and control groups, FN exhibited a moderate negative correlation with D and ADC. Additionally, OV demonstrated a negative correlation with D and ADC (Figure 4).

## Discussion

This study assessed the diagnostic efficacy of conventional MRI and IVIM for PCOS and investigated the correlation between ovarian morphological and ovarian stroma changes in PCOS patients. The findings revealed that the D* and f values of the ovarian stroma in PCOS patients were significantly increased, while the ADC and D values were significantly reduced. Additionally, ADC and D values showed a negative correlation with FN and OV. The combined use of ADC and FN values enhanced the diagnostic efficacy for PCOS.

Accurate diagnosis is essential for timely intervention and long-term health management for PCOS. US is widely used in clinical practice to evaluate OV and FN. However, US has limitations in patients with obesity or suboptimal imaging windows due to operator dependency and variability (11). By contrast, MRI provides high tissue resolution, multi-planar imaging without radiation, and has been shown to outperform US in detecting morphological features of PCOS such as OV and FN, particularly in obese or adolescent populations (12, 13). In this study, we confirmed that FN and OV form MRI exhibited good diagnostic efficiency in PCOS with AUCs of 0.85 (95% CI: 0.78–0.93) and 0.90 (95% CI: 0.84–0.96) for OV and FN, respectively.

Beyond morphological changes, studies have shown that PCOS is also associated with abnormalities in the ovarian stroma, including increased echogenicity, fibrosis, and altered blood flow (14, 15). By using IVIM technique, our findings revealed significantly reduced ADC and D values in the PCOS cases, which suggests restricted water diffusion in the ovarian stroma. This may reflect increased stromal collagen deposition. As a previous study showed that increased stromal collagen deposition on stromal fibrosis in PCOS, which hinders the diffusion of water molecules (16). Additionally, we found that FN and OV exhibited negative correlation with the D and ADC values of the ovarian stroma, indicating that stromal diffusion restriction is more pronounced in patients with greater ovarian enlargement and follicle counts.

We observed that the D* and *f* values were significantly higher in the PCOS cases, which indicates increased microcirculation perfusion and microvascular density in ovarian stroma of PCOS cases. This result is consistent with the findings of previous studies reporting increased ovarian stromal blood flow and vascularization in PCOS (7, 17). The elevated D* may reflect changes in capillary architecture or increased flow velocity (18), which possibly links to higher serum VEGF levels in PCOS (19). The increase in f value is consistent with histopathological evidence showing higher microvascular density in the ovarian stroma of PCOS patients (20).

Importantly, our study demonstrated that that combining functional imaging parameters (ADC) with morphological features (FN) significantly improved diagnostic performance for PCOS. While previous research using mono-exponential DWI achieved an area under the curve (AUC) of 0.72 with an ADC cutoff value of 1.38 (9). Our IVIM-based approach yielded an improved AUC of 0.83 with a lower ADC cutoff value of 1.104. After combination of ADC value and FN, a significant imprisonment in diagnosis of PCOS was shown with an AUC of 0.94 (95% CI: 0.90–0.98). This suggests that IVIM provides more sensitive and specific information of ovarian stroma for differentiating PCOS.

This study has several limitations. First, IVIM parameter values were measured in only a few slices of the ovarian stroma rather than across the entire ovarian stroma, which may not fully capture its heterogeneous microstructure. This limited sampling could lead to biased estimates and reduce the generalizability of the findings. Second, the study focused solely on common ovarian morphological parameters (OV and FN), excluding other potential morphological features. Furthermore, the study was conducted at a single center with a limited sample size, highlighting the need for further multi-center studies with larger sample size to validate the diagnostic value of IVIM imaging parameters in PCOS patients. Lastly, MRI also has inherent drawbacks, including higher cost, longer examination times, and reduced accessibility compared to traditional US. Therefore, MRI with IVIM may serve as a complementary tool to US in selected clinical scenarios where additional diagnostic information is required or when US is inconclusive.

## Conclusion

Clinically, our findings highlight the potential value of IVIM as a non-invasive, radiation-free imaging modality that enhances understanding of ovarian stromal alterations in PCOS. The integration of morphological and functional imaging could improve early diagnosis, support individualized risk assessment, and guide treatment decisions, especially in patients where US is limited or detailed stromal evaluation is warranted.

## Data Availability

The raw data supporting the conclusions of this article will be made available by the authors, without undue reservation.
