# Supplementary material for: Application of intravoxel incoherent motion imaging in the diagnosis of polycystic ovary syndrome
Source: Front Med (Lausanne). 2025 Jun 5;12:1513710. doi: 10.3389/fmed.2025.1513710 (PMC12176728; doi:10.3389/fmed.2025.1513710)
Supplement: Supplementary file 1 [file Table_1.docx]

Supplementary Table 1 MRI Scanning Sequences and Parameters

| Parameter | Axial T2WI | Sagittal T2WI | Coronal T2WI | IVIM-DWI |
| --- | --- | --- | --- | --- |
| Sequence | SE | SE | SE | EPI |
| Repetition Time (ms) | 5500 | 8460 | 1400 | 4700 |
| Echo Time (ms) | 102 | 102 | 104 | 71 |
| Slice Thickness (mm) | 2.0 | 2.0 | 5.0 | 2.0 |
| Slice Interval (mm) | 0.6 | 0.6 | 1.0 | 2.6 |
| Field of View (mm) | 353 × 353 | 265 × 479 | 300 × 542 | 374 × 268 |
| Matrix | 320 × 320 | 320 × 320 | 320 × 320 | 86 × 120 |
| Acquisition Time (s) | 106 | 84 | 216 | 416 |

SE: spin echo; T2WI: T2-weighted imaging; IVIM-DWI: intravoxel incoherent motion- diffusion-weighted imaging; EPI: echo-planar imaging.

Supplementary Table 2 Subgroup analysis of the PCOS cases

|  | Control (N=60) | HA+OA (N=22) | HA+OA+PCO (N=22) | OA+PCO (N=12) | HA+PCO (N=2) | P-value1 | P-value2 | P-value3 | P-value4 |
| --- | --- | --- | --- | --- | --- | --- | --- | --- | --- |
| Age (y) | 26±2.5 | 25±3.9 | 25±5.3 | 25±5.4 | 20±8.4 | 0.91 | 0.997 | 0.995 | 0.184 |
| BMI (kg/m^2^) | 20±3.0 | 24±5.2 | 25±4.5 | 24±3.6 | 24±5.0 | 0.001 | <0.001 | 0.021 | 0.676 |
| OV (cm) | 7.0±2.5 | 10.3±4.8 | 13.8±4.1 | 12.9±3.1 | 11.3±2.1 | 0.002 | <0.001 | <0.001 | 0.439 |
| FN | 14±4.6 | 21±8.5 | 32±8.6 | 30±5.0 | 24±9.9 | <0.001 | <0.001 | <0.001 | 0.255 |
| ADC (×10^-3^ mm^2^/s) | 1.26±0.23 | 1.04±0.15 | 0.98±0.14 | 0.95±0.12 | 1.19±0.39 | <0.001 | <0.001 | <0.001 | 0.989 |
| D (×10^-3^ mm^2^/s) | 1.36±0.19 | 1.23±0.13 | 1.16±0.13 | 1.17±0.12 | 1.37±0.37 | 0.024 | <0.001 | 0.005 | 1 |
| D* (×10^-3^ mm^2^/s) | 9.48±1.72 | 10.2±1.00 | 10.9±1.00 | 10.0±0.90 | 11.1±2.10 | 0.255 | 0.002 | 0.741 | 0.508 |
| f | 0.12±0.06 | 0.1±0.1 | 0.15±0.04 | 0.1±0.03 | 0.1±0.01 | 0.115 | 0.282 | 0.695 | 0.997 |
| LH (IU/L ) | 5.5±2.9 | 13.2±9.8 | 13.5±6.54 | 9.6±6.5 | 8.7±4.1 | <0.001 | <0.001 | 0.198 | 0.941 |
| FSH (IU/L ) | 8.0±1.6 | 6.8±2.2 | 6.2±1.74 | 5.8±1.5 | 3.3±1.1 | 0.059 | 0.001 | 0.001 | 0.003 |
| T (ng/mL) | 0.4±0.1 | 0.9±0.5 | 1.0±0.4 | 0.4±0.1 | 0.8±0.1 | <0.001 | <0.001 | 1 | 0.532 |
| LH/FSH | 0.7±0.4 | 1.9±1.1 | 2.2±1.2 | 1.6±1.0 | 2.5±0.3 | <0.001 | <0.001 | 0.01 | 0.031 |
| PCO-US |  |  |  |  |  |  |  |  |  |
| Negative | 50 (83.3%) | 22 (100%) | 0 (0%) | 0 (0%) | 0 (0%) | 0.096 | <0.001 | <0.001 | 0.043 |
| Positive | 10 (16.7%) | 0 (0%) | 22 (100%) | 12 (100%) | 2 (100%) |  |  |  |  |
| Hyperandrogenism |  |  |  |  |  |  |  |  |  |
| Negative | 51 (85.0%) | 0 (0%) | 0 (0%) | 12 (100%) | 0 (0%) | <0.001 | <0.001 | 0.339 | 0.031 |
| Positive | 9 (15.0%) | 22 (100%) | 22 (100%) | 0 (0%) | 2 (100%) |  |  |  |  |
| Oligoanovulation |  |  |  |  |  |  |  |  |  |
| Negative | 51 (85.0%) | 0 (0%) | 0 (0%) | 0 (0%) | 2 (100%) | <0.001 | <0.001 | <0.001 | 1 |
| Positive | 9 (15.0%) | 22 (100%) | 22 (100%) | 12 (100%) | 0 (0%) |  |  |  |  |

All compared to the controls. P-value1 for HA+OA, P-value2 for HA+OA+PCO,P-value3 for OA+PCO, and P-value4 for HA+PCO
